# Airline Cabin Crew Team System’s Positive Evaluation Factors and Their Impact on Personal Health and Team Potency

**DOI:** 10.3390/ijerph181910480

**Published:** 2021-10-06

**Authors:** Youkyung Ko, Hwaneui Lee, Sunghyup Sean Hyun

**Affiliations:** 1 School of Tourism, Hanyang University, 222, Wangsimni-ro, Seongdong-gu, Seoul 04763, Korea; y5ukyung@hanyang.ac.kr; 2 Department of Hotel and Tourism Management, Kyungmin University, Uijeongbu 11618, Korea; honey2000@hanmail.net

**Keywords:** airline cabin team system, sense of belonging, mutual support, communication, motivation, work flexibility, job satisfaction, team potency, mental health

## Abstract

Recently, many airline companies have trialed introducing team systems to manage crew members and enhance competitiveness systematically through the efficiency of manpower operation. Cabin crew members share in a sense of unity when spending time with team members outside of work hours. Cabin crews must be able to resolve unexpected issues—fires, aircraft defects, medical emergencies, and sudden airflow changes—quickly and accurately. As unexpected issues may result in major accidents, it is crucial that cabin crew members can take responsibility for passenger safety and offer satisfactory services to customers. Furthermore, most cabin duties require cooperation and are highly interdependent; thus, respect and teamwork are essential. This empirical study aims to identify and examine the positive factors of the team system used to evaluate causalities in job satisfaction, team potency, and mental health. The research model is developed based on a theoretical review, focusing on five positive factors—sense of belonging, mutual support, communication, motivation, and work flexibility—and dependent variables: job satisfaction, team potency, and mental health. Sense of belonging, communication, and work flexibility significantly affected team potency along with job satisfaction. This study has practical implications, providing guidance for the sustainable development of team systems for airline crew management.

## 1. Introduction

In recent decades, teamwork has given new opportunities to companies. Teams have been essential in resolving problems and have contributed to the development of companies [1]. Teamwork has a significant role in society. Talented executives continuously try to introduce and develop team systems because of their synergy effects and advantages. The airline industry is no exception. After the introduction of the cabin team system in Korean Air in 1986, the company has maintained and operated the system to date. Team systems’ main purpose in airline companies is to manage many cabin crews systematically and enhance competitiveness through the increased efficiency of HR (human resource) management [2]. Specifically, cabin crews (including cabin crew in trains or tourism cruise ships) work in a unique environment where there is no direct contact with the outside world. Thus, it is necessary for cabin crew members to respond immediately to various situations, including aircraft defects, medical emergencies, and sudden changes in turbulence. In the occurrence of an unexpected situation, a skilled and cooperative team can resolve problems immediately and accurately. A positive relationship between team members and co-workers is crucial. This relationship is even more necessary considering cabin crews’ uncommon work environment and the high expectations for customer satisfaction. Most of the cabin crews’ duties are highly interdependent, and such a work environment requires special respect among members and a focus on teamwork to generate satisfactory results. In such environments, mutual support among employees is the key to overcoming difficult situations, which is why teamwork is vitally important.

A study determining the advantages of the team system has not yet been extensively conducted, and discussions on the topic are ongoing for airline companies. In other words, significant advantages of the team system have been identified; however, these advantages have not been proved to enhance employee performance. Therefore, arguments for and against team systems are still ongoing. The purpose of this study is (1) to systematize and establish the five factors of the team system, (2) to determine the factors that bring out the maximum crew member satisfaction, and (3) to review the dynamic toward the enhancement in team ability through the team system. Ultimately, this research will provide theoretical and practical guidance for airline team systems in line with sustainability and usage, shedding light on whether these systems are relevant for the future.

## 2. Conceptual Framework

### 2.1. Advantages of the Airline Cabin Team System

The team system is ultimately designed to help companies deal with internal problems by boosting productivity, thus increasing flexibility in their business. This allows and assists team members with seeking and solving problems in collaboration with others. Here, teams achieve better results than individuals in limited job roles [1]. In addition, teams encourage participation, providing challenges and fulfillment for their members, enabling organizations with teams to create flexible, efficient, and profitable high-performance organizations. Therefore, organizations can achieve high performance by appropriately utilizing the benefits of the team system, such as team members respecting and helping each other when necessary rather than trying to outperform each other, as well as sharing and developing ideas and skills with other team members. With this emphasis on the importance of team systems, many companies are gradually trying to develop them. Airlines are no exception to this phenomenon. Cabin crews set common goals to help the team achieve good results and plan strategies, cooperating toward these common goals. In this study, we present the advantages of the airline cabin team systems for in-flight duties where teamwork, among other things, is essential.

#### 2.1.1. A Sense of Belonging

Maslow [3] defines a sense of belonging as human beings’ basic social need to be recognized by others, and Hagerty et al. [4] define it as an experience of personal involvement that makes individuals feel they are an integral part of their social system or environment.

The importance of research into the sense of belonging has been highlighted by the discovery that such a sense in organizational life has a significant impact on individual behavior [5]. People need personal contact or frequent interaction with others to create interpersonal relationships or form bonds. Additionally, intimate social contact gives a sense of belonging, which provides satisfaction that cannot be felt in interactions with strangers or new acquaintances [6]. Thus, it is expected that cabin crew team members flying with other members who know each other well and have close relationships will feel a greater sense of belonging and be more satisfied than others who do not fly as a team.

Members whose sense of belonging helps them develop their organization will often devote themselves to their organization while making personal sacrifices [7]. As crew members spend time with their team members, they become familiar with each other and emotionally attached, and they develop a stronger sense of belonging to the organization and having a shared experience. Studies of a sense of belonging in the workplace show that members (a) learn to be part of a group, (b) connect with and include peers, (c) choose to be recognized for doing meaningful work, (d) find themselves deeply involved in their job, and (e) understand time and work well while considering their relationships with other people [8]. Through a sense of belonging, team members can rely on each other, find solutions to various personal problems as well as work-related problems, and gain greater satisfaction with their work.

Winter-Collins and McDanie (2000) conducted a survey on newcomer nurses who had been employed within 18 months of obtaining a nurse (RN) license to examine the relationship between newcomer nurses’ sense of belonging and job satisfaction. It was suggested that the high sense of belonging of newcomer nurses is related to high job satisfaction [5]. Based on the theoretical background, the following three hypotheses are derived:

**Hypothesis** **1-1.** *The cabin crew’s sense of belonging has a significant effect on job satisfaction.*

**Hypothesis** **1-2.** *The cabin crew’s sense of belonging has a significant effect on team potency.*

**Hypothesis** **1-3.** *The cabin crew’s sense of belonging has a significant effect on mental health.*

#### 2.1.2. Mutual Support

Hoegl and Gemuenden [9] define mutual support as helping and supporting each other in performing duties. Baker et al. [10] also describe mutual assistance as providing feedback and coaching between team members to improve performance or completing tasks for teammates when needed. Mutual support is an integral part of a team’s effective performance. Mutual backing in a team encourages continuous interactions of support, thereby achieving the team’s goal of cohesion with a sense of belonging. In a mutually supportive working environment, workers try to complement each other to reach a consensus on important issues, and, in a collaborative atmosphere, workers can experience mutual respect when discussing proposals; these contributions often lead to meaningful development [11].

The team environment provides employees with opportunities to share knowledge and learn from others. These opportunities thereby improve employee productivity and overall team performance, which leads to increased levels of shared learning and productivity through team members’ collaboration [12]. Therefore, cabin crew members who are willing to share the know-how regarding how to perform their duties skillfully through mutual support with their team members create more effective and skilled teammates and teams as a whole.

The interdependence of crew members is magnified when unusual or urgent situations arise. Flight attendants must identify problems, develop plans according to risk levels and time limits, and decide the course of action. This decision process increases the need for mutual assistance among crew members [13]. Additionally, it is important to remember that cabin crews who work in the same work environment are more dependent on their members than other departments because these team members work jointly within a set flight time in the limited, closed space of an aircraft. Since mutual support facilitates the integration of team members’ expertise, it is an important aspect of team collaboration [9]. Furthermore, one of the important elements of a team system is the building of trust and a positive atmosphere; team members should help, respect, and encourage other team members rather than competing, which creates distrust and tension [14]. For these reasons, teams have the potential to share more knowledge, skills, and experience through mutual support than individuals can do alone, which is expected to impact the job positively. The following hypotheses are proposed regarding the relationship among mutual support, job satisfaction, team potency and mental health:

**Hypothesis** **2-1.** *The mutual support of cabin crew members has a significant effect on job satisfaction.*

**Hypothesis** **2-2.** *The mutual support of cabin crew members has a significant effect on team potency.*

**Hypothesis** **2-3.** *The mutual support of cabin crew members has a significant effect on mental health.*

#### 2.1.3. Communication

The dictionary meaning of communication is “people exchanging information such as thoughts and feelings with each other; consisting of words, writings, other sounds, facial expressions, and gestures”. Fiske [15] defines communication as social interaction through messages, and Dimbleby and Burton [16] describe it as being able to express one’s thoughts and opinions and understand others’ opinions.

Communication is an important factor to consider for team management and talent development because, during effective communication, information exchange occurs which allows team members to acquire and create various kinds of information [17]. Additionally, effective communication based on the knowledge of various team members facilitates information exchange and generates new knowledge and insights [18]. Communication is also emotionally shared by dedicated employees and managers and is a powerful catalyst for building and maintaining trust.

Kanki [19] emphasizes the importance of communication in achieving safe and efficient flight operations and work goals because communication among crew members in the flight preparation process, including briefings to plan the overall flight beforehand, can significantly positively impact the flight through to the landing stage. In addition, it is said that communication serves as a behavioral indicator for decision making, problem solving, and resource and workload management. In-flight communication allows flight crews to know if work is being carried out as planned and according to normal procedures and if problems are occurring. When large or small problems occur during the flight, accurate information delivery through communication can play a key role in solving these problems.

King, Lahiff and Hatfield (1988) conducted a study on undergraduate students who had been working for more than 5 months and suggested that there was a significant positive relationship between communication and job satisfaction [20].

Based on the theoretical background, we formulated the following hypotheses stating that there are significant relationships among communication, job satisfaction, team potency, and mental health.

**Hypothesis** **3-1.** *Cabin crew communication has a significant effect on job satisfaction.*

**Hypothesis** **3-2.** *Cabin crew communication has a significant effect on team potency.*

**Hypothesis** **3-3.** *Cabin crew communication has a significant effect on mental health.*

#### 2.1.4. Motivation

Bartol and Martin [21] define motivation as a force that invigorates behavior, gives direction to action, and triggers a tendency to persist. Additionally, Conrad, Ghosh, and Isaacson [22] describe motivation as an inner force that drives individuals to achieve individual and organizational goals.

Employee motivation is one of the key drivers of a company’s success in today’s competitive environment, so it is important for managers to find out what motivates employees to achieve goals and eventually achieve job satisfaction [23].

In organizational management, members must be motivated and energetic enough to realize an organization’s objectives, focus clearly on what they need to achieve, and influence them so that they are willing to devote their energy long term [24]. Motivation positively influences job satisfaction by encouraging enthusiasm among team members to perform their roles in the team [25]. Integrating the theoretical background, the following hypotheses can be derived:

**Hypothesis** **4-1.** *Cabin crew motivation has a significant effect on job satisfaction.*

**Hypothesis** **4-2.** *Cabin crew motivation has a significant effect on team potency.*

**Hypothesis** **4-3.** *Cabin crew motivation has a significant effect on mental health.*

#### 2.1.5. Work Flexibility

As the global business environment becomes increasingly dynamic, organizations have sought to increase flexibility to respond quickly to changing environments [26] and chaotic and unpredictable environments where continuous globalization and rapid advances in information technology shorten product lifecycles. Jonsson [27] describes flexibility as the tendency of an actor (business, organization, employee) or organizational system to exhibit a change in the desired activity or condition in response to changing circumstances. Furthermore, Lu et al. [28] define it as having autonomy and personal control over his or her work schedule and routine.

One of the ways to increase flexibility is to empower workers to make decisions and perform their duties [29]. During team flights, it can be relatively easy to apply functional flexibility such as rotating assigned crew duties to improve the abilities of team members to perform multiple tasks. For the efficient use of human resources and the convenience of customers, the speed of work, time of service, method of service, and so on may be adjusted autonomously within the scope of the company’s regulations. Furthermore, by directly monitoring the method or quality of services, it can be relatively easy to exercise functional work flexibility. The flexible working environment created by team members has a positive impact on the team [30].

Furthermore, increased work flexibility means that the set work time and service methods can shift so that a crew’s work can be more closely aligned with the customer’s needs, thereby enhancing the company’s profit and competitiveness. Therefore, it can be expected that employee satisfaction will be greatly increased in a cabin crew culture that can implement work flexibility based on the needs of passengers.

Annakis, Lobo, and Pilla (2011) studied the effect of work flexibility on job satisfaction in the customer service officer group (CSR) of an Australian call center. As a result of the study, it was found that the work flexibility was directly and positively related to job satisfaction [31]. We formulated the following hypotheses stating that significant relationships existed among the three constructs.

**Hypothesis** **5-1.** *Cabin crew work flexibility has a significant effect on job satisfaction.*

**Hypothesis** **5-2.** *Cabin crew work flexibility has a significant effect on team potency.*

**Hypothesis** **5-3.** *Cabin crew work flexibility has a significant effect on mental health.*

### 2.2. Job Satisfaction

While scholars’ definitions of it vary, generally, job satisfaction is the positive feeling and attitude that individuals feel about their job. Locke [32] defines job satisfaction as a positive emotional state resulting from satisfying or promoting the value of one’s job. Spector [33] states that job satisfaction is the extent to which employees like their jobs, and it directly affects the well-being of the employees’ lives.

Employees gain job satisfaction when their expectations and values are met at work [34]. An increased level of employee job satisfaction leads to pride and loyalty in the organization, which contributes to improving the productivity of the organization [35].

From the customers’ perspective, employee job satisfaction is a critical factor in the quality of service provided to the customers, and high job satisfaction is directly or indirectly communicated to the customers during their interactions with an employee and leads to high performance [36]. Therefore, an entity must strive to improve not only customer satisfaction but also employee satisfaction. Higher levels of job satisfaction among employees lead to pride in and loyalty to the organization [33], thus improving the productivity of the organization with a responsible and active attitude.

Pincus (1986) investigated the effect of communication satisfaction on job performance and job satisfaction among 327 nurses working in hospitals. As a result, they found a significant positive relationship between communication satisfaction and job satisfaction within the nurse’s organization [37,38]. The sixth and seventh hypotheses in this work are as follows:

**Hypothesis** **6.** *The job satisfaction of cabin crew members has a significant effect on team potency.*

**Hypothesis** **7.** *The job satisfaction of cabin crew has a significant effect on mental health.*

### 2.3. Team Potency

Team potency refers to the trust team members have in their collective competencies [39]—a common belief that their teams can perform certain tasks [40,41]. Team potency can be an indicator of a company’s performance, as it can be evaluated not only by how well the team actually performs but also by the members’ confidence and belief that it will continue to perform well.

In an empirical study by del Barco, Mendo-Lázaro, Felipe-Castaño, del Río, and Fajardo-Bullón [42], it was shown that teamwork affects team competency when a team works collaboratively. In other words, members increase their confidence in the team (team potency) and their ability to complete tasks successfully when they learn together, help each other, and satisfactorily solve problems that arise while performing teamwork.

Duffy and Shaw (2000) conducted a study on the relationship of team effectiveness and team performance, and the results showed that teams with high team efficacy performed better than those with low team efficacy [43].

### 2.4. Mental Health

Mental health is an important factor in determining quality of life [44]. In line with an increasing understanding of the importance of mental health, many companies in advanced countries are seeking to improve their employees’ mental health by introducing EAPs (Employee Assistance Programs). EAPs can help to manage employees’ mental health by controlling their job stress or helping employees manage this stress through professional psychological counselors or outside agencies within the program [45].

Mental health is defined by the World Health Organization (WHO) [46] as “a state of well-being in which the individual realizes his or her own abilities, can cope with normal stresses of life, can work productively and fruitfully, and is able to make a contribution to his or her community.”

Recently, people have become aware that job stress from work environments can negatively affect mental health, adversely impacting not only the workers’ own quality of life but also colleagues, employers, companies, families, and the economy, so many studies have been conducted on this topic [47].

Workers who provide commercialized services based on interpersonal relationships and emotional support are relatively more exposed to the risk of mental stress; so, repeated mental stress, exhaustion, and emotional dissonance can lead to serious mental health problems such as depression or panic disorders. Cabin crew members are also part of this emotional workforce, and they are responsible for providing services to many customers while being responsible for their safety. Thus, it is necessary to look at employees’ mental health and how it is affected by the mental stress generated from work.

## 3. Research Methods

### 3.1. Study Models and Hypotheses

**Figure 1:**
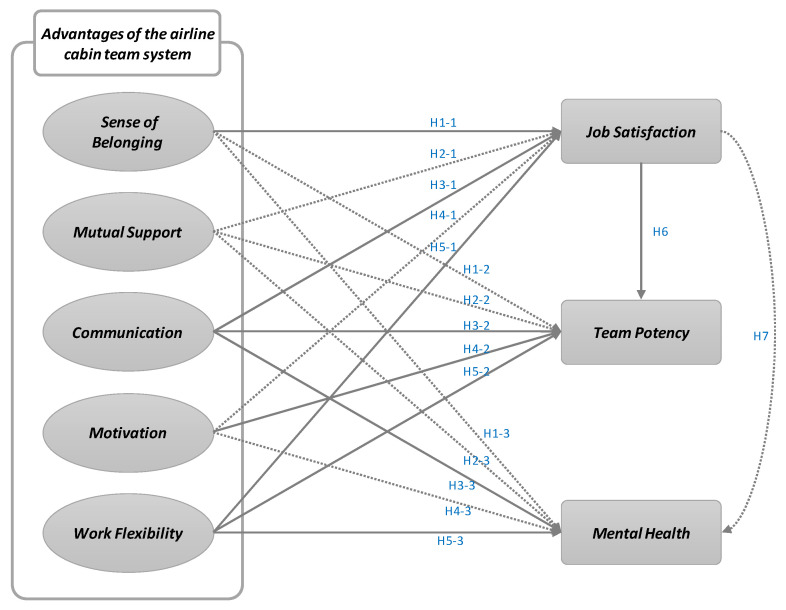
Research model.

This study was conducted on domestic airline cabin crew members. It was intended to establish the relationship between leading and trailing variables of positive factors of the cabin crew team system. Seventeen hypotheses were derived based on theoretical and empirical backgrounds and research models were derived by integrating them. Figure 1 presents the research model and hypotheses.

### 3.2. Measurement

To develop the measurement tools in this study, five key variables that benefit airline cabin team systems were identified: (1) sense of belonging was measured with 4 items employed by Mael and Ashforth (1992) [48], (2) mutual support was measured with 3 items adapted by Lechler (2001) [49], Baruch-Feldman et al. (2002) [50] and Bridges, Sherwood, and Durham (2014) [51], (3) communication was measured with 4 items adapted by Lee (2000) [52] and Jeon (2008) [53], (4) motivation was measured with 5 items adapted by Ryu (2002) [54] and Cho (2017) [55], (5) work flexibility was measured with 5 items employed by Bhattacharya, Gibson, and Doty (2005) [55] (6) job satisfaction was measured with 4 items employed by Ryu (2002) [54], Cho (2017) [55], and Bhattacharya, Gibson, and Doty [56], (7) team potency was measured with 4 items adapted by Lester, Meglino, and Korsgaard (2002) [57], del Barco et al. (2017) [42], and (8) mental health was measured with 5 items adapted Veit and Ware (1983) [58].

The equivalent scale used a 5-point Likert scale (1 = “strongly disagree”; 5 = “strongly agree”) where a value of 1 is the respondent’s strong negative view and a value of 5 implies the respondent’s strong positive view.

### 3.3. Collecting Data and Analysis Method

#### 3.3.1. Collecting Data

For the validity of the measurement items of this study, a preliminary survey was conducted on cabin crew (*n* = 30) prior to this survey. The survey was conducted based on a final review by professors, supplementing the problems raised through the preliminary survey. The survey was conducted from 1 January to 1 April 2021 (three months) on cabin crew members currently working for a domestic airline and belonging to a team, and the data collection was conducted by distributing a total of 300 questionnaires through social media.

#### 3.3.2. Analysis Method

The statistical analysis methods used for the analysis of collected data were as follows. First, we conducted frequency analysis and descriptive statistical analysis to understand the general characteristics of the subject. Second, we conducted a Confirmatory Factor Analysis (CFA) to assess the feasibility of the latent variables and the observational variables that make up the measurement model of the present work, verified the composite reliability (CR) and average variance extracted (AVE), and confirmed the Cronbach’s α coefficient to verify the reliability of the measurement tool. Third, we conducted technical statistical analysis to identify the advantages of airline cabin team systems, job satisfaction, mental health, and team competency levels, and to determine whether normality assumptions were met. Additionally, average, standard deviation, skewness, and kurtosis were identified. Fourth, we conducted a structural equation model (SEM) to verify structural relationships between airline cabin team systems, job satisfaction, mental health, and team competencies. Fifth, we carried out bootstrapping to verify the indirect effects of the airline’s cabin team system on mental health and team capabilities by mediating job satisfaction. For statistical analysis, IBM SPSS 25 and AMOS 25 were utilized, and statistical significance was determined based on a 5% level of significance.

## 4. Research Results

### 4.1. Characteristics of the Sample

Table 1 shows the demographic profile of the survey participants. First, regarding the respondents’ gender, 234 (86.7%) were women and 36 (13.3%) were men. The average age was 33.44 years, with five people aged 20–25 (1.9%), 92 (34.1%) aged 26–30, 84 (31.1%) aged 31–35, 64 (23.7%) aged 36–40, 18 (6.7%) aged 41–45, and seven older than 46 (2.6%). The number of married participants was 160 (59.3%), with 110 unmarried participants (40.7%). Fifty-five were junior college graduates (20.4%), 198 were college graduates (73.3%), 11 were graduate students (4.1%), and six were graduate school graduates (2.2%). The average number of working years was 8.5, with seven people (2.6%) having worked for under 2 years, 55 (20.4%) having worked between 2 and 5 years, 97 (35.9%) having worked between 5 and 10 years, 81 (30.0%) having worked between 10 and 15 years, and 30 people having worked over 15 years (11.1%). The positions of the participants included 134 flight attendants (49.6%), 91 vice pursers (33.7%), 38 pursers (14.1%), five senior pursers (1.9%), and two chief pursers (0.7%). Regarding annual salaries, two people earned under 20 million won (0.7%), 50 people earned between 20 million and 35 million won (18.5%), 114 people earned between 35 million and 50 million (42.2%), and 104 people (38.5%) had salaries of more than 50 million won. The positions of the participants were 13 team leaders (4.8%), 12 vice team leaders (4.4%), and 245 team members (90.7%).

### 4.2. Verification of Reliability and Feasibility of Measurement Variables

In this work, before identifying structural relationships between airline cabin crew systems, job satisfaction, mental health, and team competence, CFA was conducted to verify the validity of latent and observational variables that make up the structural model.

The SEM of this study establishes a total of five perceptions—belonging, mutual support, communication, motivation, and work flexibility—as independent variables, job satisfaction as a parameter, and mental health and team competence as dependent variables. In short, the model consisted of eight latent variables.

**Figure 2:**
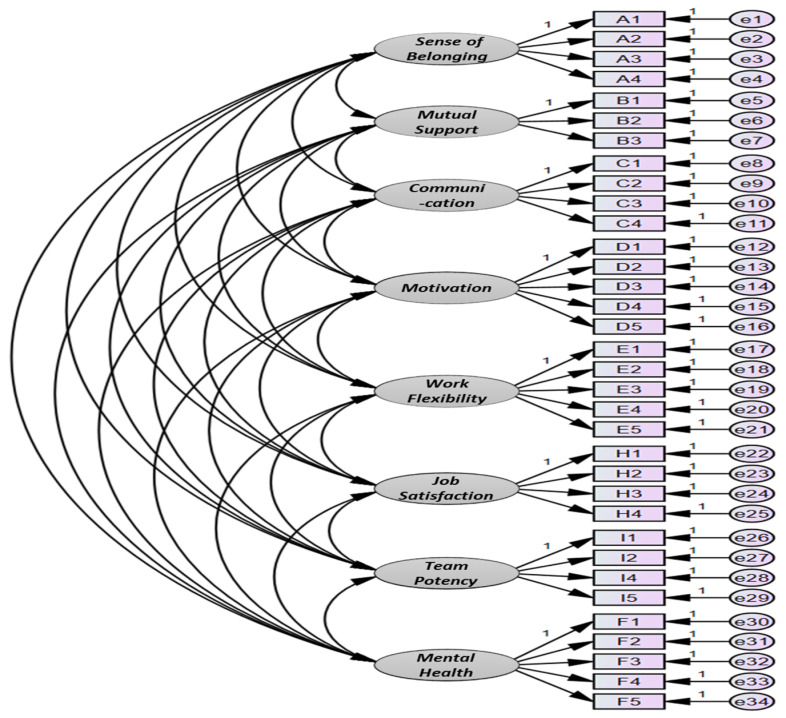
Confirmative factor analysis model.

Of the eight latent variables, belonging, communication, job satisfaction, and team competence consisted of four items; mutual support consisted of three items; and motivation, work flexibility, and mental health consisted of five items. The measurement model constructed to proceed with this identifying factor analysis is shown in Figure 2.

#### 4.2.1. Confirmative Factor Analysis and Reliability Analysis

**Figure 3:**
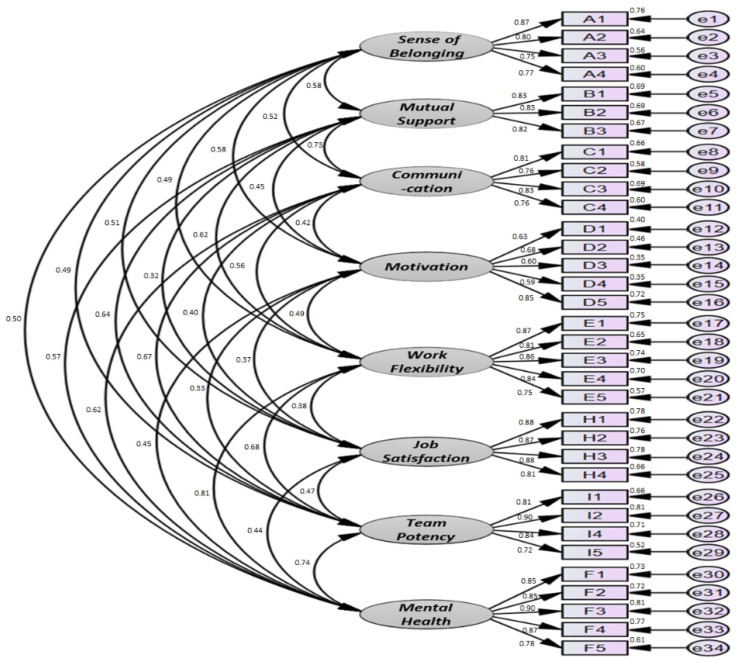
Confirmative factor analysis results.

Confirmative factor analysis was conducted to determine the feasibility of the latent variables and the observational variables that make up the measurement model, and the results are presented in Table 2 and Figure 3. The confirmative factor analysis showed that the factor load was between 0.590 and 901. All factor loads show significant results above 0.50 (*p <* 0.001). Observers can be seen as a good reflection of that latent variable.

Meanwhile, the comparative fit index (CFI) of the model was found to be good at 0.90 or higher (measurement model: 0.953), the Tucker–Lewis index (TLI) was 0.90 or higher (0.947), and the root mean square error of approximation (RMSEA) was less than 0.08 (met the baseline) [59]. In short, the fit of the model can be considered good, and the composition of the measurement model can be considered reasonable.

Furthermore, we confirmed Cronbach’s coefficients to determine the intrinsic consistency of items within the constructed factors. The Cronbach’s coefficient of all variables had a high value of 0.70 or higher (see Table 2), meaning that the reliability of the measurement tool is good.

#### 4.2.2. Convergence Feasibility Verification

Before proceeding with the SEM analysis, various validations must be conducted. The correlation of the observers who make up the latent variables must be high enough to determine whether they converge well.

Convergence feasibility can be verified by CR and AVE; the results are shown in Table 3. In general, convergence feasibility is considered good when synthetic confidence is 0.70 or higher and the AVE is 0.50 or higher [59]. All variables were considered to have convergence feasibility in this measurement model.

#### 4.2.3. Verification Validation

To verify the discriminant validity, the AVE value —the square value of the correlation coefficient—is compared. Generally, a higher AVE value of all variables than the inter-substantiation coefficient means the model has a good discriminative validity [60]. The results of the discriminant validity verification in this measurement model are presented in Table 4.

The maximum value of the correlation coefficient between the latent variables of this measurement model was found to be 0.812; the correlation coefficient between task flexibility and mental health had a square value of 0.659. However, the AVE was found to have the lowest motivation of 0.686; that is, the minimum value of the AVE value was shown to be higher than the maximum value of the correlation coefficient square between latent variables, resulting in a higher AVE value of all variables than the determination coefficient of all latent variables. Consequently, this measurement model has obtained discriminatory validity.

#### 4.2.4. Technical Statistics and Regular Verification

As a key variant of the study, descriptive statistical analysis was conducted to determine the level of belonging, mutual support, communication, motivation, work flexibility, job satisfaction, team competence, and mental health that comprise the airline’s cabin team system. All variables were measured on a 5-point scale, with an average of 3.63—mutual support averaging 4.02, communication averaging 3.72, motivation averaging 3.70, and work flexibility averaging 3.87. Job satisfaction, which is a parameter, averaged 3.20, with a team competence average of 3.66 and a mental health average of 3.58. The Table 5 provides the verification validation results. 

Meanwhile, skewness and kurtosis were identified to determine whether the data met the normality assumption. Generally, skewness is considered to satisfy the normality assumption if it is less than ±2 and kurtosis is less than ±7 [61], and data can be considered to satisfy the normality assumption if all variables meet the baseline. In other words, the distribution of data can be judged to be problematic in conducting SEM analysis.

### 4.3. Analysis of Structural Models and Hypothesis Validation

#### 4.3.1. Model Configuration

**Figure 4:**
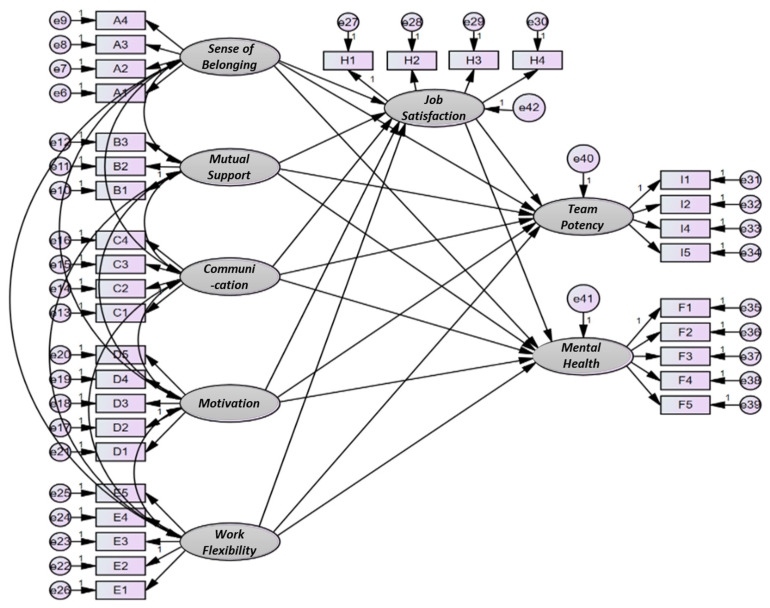
Structural equation model.

This study seeks to verify the awareness of the advantages of airline cabin team systems, job satisfaction, and structural relationships between team capabilities and mental health. Therefore, we constructed an SEM as shown in Figure 4.

#### 4.3.2. Goodness-of-Fit

The main goodness-of-fit index was identified to determine the suitability of the SEM constructed for this study, and the results are shown in Table 6. The main goodness-of-fit index, CFI, TLI, was 0.946, and RMSEA was 0.049. Both CFI and TLI were above 0.90 and RMSEA was less than 0.08, indicating that the goodness-of-fit index for the SEM is adequate.

#### 4.3.3. Structural Equation Model Analysis Results

**Figure 5:**
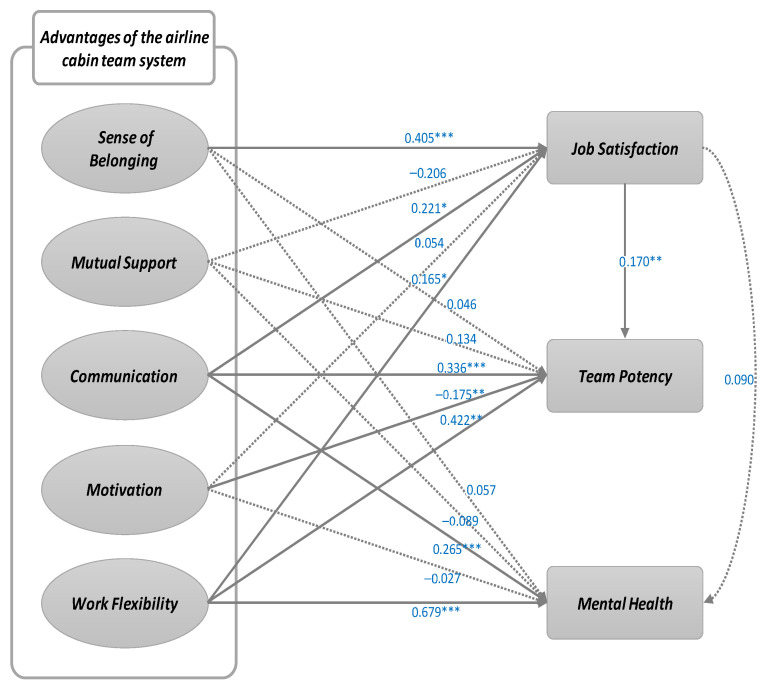
Structural equation model analysis results.

Table 5 provides the structural equation modeling results. Among the advantages of the airline’s cabin team system, the level of job satisfaction was verified as high, the level of communication and work flexibility was verified as high, and the higher the level of job satisfaction, the better the team. As a result, hypotheses H1-1, H3-1, H3-2, H3-3, H5-1, H5-2, H5-3, and H6 were supported (Figure 5).

## 5. Discussion and Implications

This study analyzed which factors of advantages of the airline cabin crew system positively impact cabin crew members of domestic airlines who operate in a team system to effectively improve organizational performance through a systematic and established team system. First, based on prior research, scholars have identified the positive factors of the airline cabin crew system and how they affect crew satisfaction as well as how they affect team competence and crew members’ mental health.

Among the key variables in this study, cabin crew members’ sense of belonging, communication, and work flexibility have positively affected job satisfaction. Mutual support and motivation have been determined to be insignificant.

The statistical analysis supported H1-1, which suggested that a sense of belonging affects job satisfaction (β = 0.405, *p <* 0.001). An interpretation here is that working with colleagues who feel more similar as team members creates more job satisfaction than in other cases. In an existing prior study [6], nurses reported being happier or more satisfied when working as a team. This current study extended prior research by applying existing findings to the aviation industry, revealing that the airline team system improves employee job satisfaction through the medium of belonging. Therefore, it is necessary to actively implement support policies that encourage team joint activities or provide education or programs for the team to boost the team’s sense of similarity and enhance the team’s sense of belonging.

Furthermore, the connection between cabin crew communication and job satisfaction showed a definite (+) significant result (β = 0.221, *p <* 0.05). Accurate information delivery is especially important for safety, which is essential in decision making, problem solving, and resource and workload management. Additionally, because team members communicate a lot with each other, communication should be easy to understand, thus reducing communication misunderstandings to deliver efficient information. A prior study [20] shows that there is a consistent and strong positive relationship between communication and job satisfaction among undergraduates who worked jobs for more than five months. Based on the results of this study, domestic airlines also need to actively utilize and develop the Crew Resource Management program to enhance communication between flight attendants and cabin crew, leading to safer flights.

H5-1, which suggested that work flexibility affects job satisfaction in the team system, was supported (β = 0.165, *p* < 0.05). This means that during team flights, it is relatively easy to understand a teammates’ work and their required duties; so, flexibility increases job satisfaction by letting team members actively participate in team decision making and increasing participatory behavior through flexibility or task integration, such as helping or taking each other’s place while working. In a prior study showing the correlation between work flexibility and job satisfaction [31], an interview with the Australian Call Center’s Customer Service Group found that work flexibility was directly and positively related to job satisfaction.

Mutual assistance is defined as team members’ willingness to provide help when others need it, such as helping a team member when they make mistakes. However, the research suggests that this may not be positively correlated to a team’s competence and efficiency if support is continuously given in one direction only (i.e., if one member of the team continuously fails to perform their duties).

Mutual assistance and support in teams continue to build interest, trust, and consideration among team members, and furthermore, they allow for the acceptance of others’ views and suggestions. This enables teams to work toward overcoming challenges and creates various helpful relationships in which effective mutual assistance can occur. Here, team members should be willing to help and seek help when needed. For example, there should be support for team members who are unable to perform their roles or systems to help other team members correct mistakes they make in performing their roles. This suggests that flight tasks, unlike other common tasks, may cause reduced job satisfaction, as gaps or mistakes in one role may result in an increase in other team members’ workloads, as all assigned objectives must be achieved within a given time, rather than individual tasks and timeframes.

Cabin crew motivation aims to increase productivity by enhancing cohesion or integration among team members and motivating individual efforts to focus on work and improve individual performance. However, this can lead team members to compare themselves with other team members in terms of performance, self-development, management, work knowledge, acquisition of skills, and recognition from superiors. Competitiveness is also created by the cabin crew’s evaluation system, which consists of relative evaluations within the team. In this instance, motivation may not have positive effects.

## 6. Conclusions

First, cabin crew members’ sense of belonging did not correlate significantly with team competence. A sense of belonging is defined as the feeling that emerges when team members spend a lot of time together and try to build close and positive relationships. While this may significantly impact individual crew members’ behavior, this was not seen in team competence.

Second, regarding the significance of the variables’ connections to competence, cabin crew communication and work flexibility were significantly correlated with team competence, but there was no significant connection with sense of belonging, mutual support, or motivation. H3-2, which suggested that communication affects team competence, was supported (β = 0.336, *p <* 0.001). Here, communication has a significant impact on team competence because it allows smooth exchanges between team members during team flight, avoiding ambiguity-induced functional conflicts and increasing the clarity of goals and processes.

The path from motivation to team competence showed significant negative results (β = −0.175, *p <* 0.01). People are motivated to do things they feel are likely to lead to valuable rewards. This survey studied cabin crew members who had spent a year with their team members and worked toward both individual and team performances. Results show that compared to the individual performances of each team member, the team performance is brought down by other team members. This can cause damage to individual team members. Competition among team members is a high stressor, which suggests that individualistic tendencies are more prominent than a willingness to consider and cooperate with other team members, indicating that this factor may not positively influence team competence.

Third, the job satisfaction of cabin crew members has been shown to significantly impact team competence (β = 0.170, *p <* 0.01). How satisfied the cabin crew is with their team, their role, and their relationship with their team members will influence team members to have a positive or negative view of their team, which significantly affects the team’s goals and performance. Members with high job satisfaction perform responsibly and actively, indicating that when job satisfaction increases, team members’ trust in their collective competence increases.

Fourth, the verification of the path to mental health showed that the paths from belonging, mutual support, and motivation to mental health and job satisfaction were not significant, but the path from communication to mental health was (+) significant (β = 0.265, *p <* 0.001). The pathway from work flexibility to mental health also showed significant (+) results (β = 0.679, *p <* 0.001). In other words, the better the perception of communication and work flexibility, the better the mental health of team members.

Finally, after verifying the indirect effects of the sense of belonging, mutual support, communication, motivation, and work flexibility, which are all advantages of airline cabin crew team systems, the indirect effect of the sense of belonging on team competence mediated by job satisfaction was (+) significant (β = 0.028, *p <* 0.05). In other words, the more positive the sense of belonging, communication, and work flexibility, the higher the job satisfaction, and the higher the job satisfaction, the higher a team’s capability.

In terms of work flexibility, the path to task satisfaction showed a definite (+) significant result (β = 0.422, *p <* 0.001). Duty can be flexibly assigned to match team members’ abilities or aptitude and adjusted to improve their performance. Moreover, in many ways, autonomously adjusting the quality or method of service within the company’s regulations improves the efficiency of tasks.

Next, belonging, mutual support, communication, motivation, and work flexibility, which are advantages of the airline cabin crew system, showed non-significant effects on mental health by mediating job satisfaction. In other words, communication and task flexibility have been proven to have a direct and significant (+) mental health impact without mediating job. As a result, among the five positive factors of the cabin crew system, belonging, satisfaction, communication, and work flexibility maximized cabin crew satisfaction, while high job satisfaction was verified to have a positive impact on team competence.

The theoretical implications of this study are as follows. Among the major variables of this study, cabin crew’s sense of belonging, communication, and work flexibility had a positive effect on job satisfaction, and mutual support and motivation were not significant. These results were found to support previous studies such as [5,20,31].

The practical implications of this study are as follows. In order to increase the team’s sense of belonging in the future, airlines should actively operate support policies such as supporting for team joint activity expenses or opening a team training program to strengthen the ties of team members. Since communication has a positive effect not only on the job satisfaction and team competency of cabin crew but also on individual mental health, airlines need to prepare a plan to effectively deal with the communication of flight attendants. Through this study, it was verified that high job satisfaction has a positive effect on team competency. Airlines should make good use of the team system to maximize the cabin crew’s job satisfaction and enhance their team capabilities.

## 7. Limitations and Recommendations for Future Research

This study identified the advantages of the cabin crew team system, outlining how crew satisfaction could be increased, which would in turn increase team capability and mental health. Here, it is important to present a limitation of this study. This survey was conducted during the COVID-19 pandemic, where cabin crew members took a long leave of absence. So, subjective thoughts and feelings about the team are likely to be diluted, and job satisfaction may be different.

In terms of future research directions, based on the literature and empirical studies, this study verified the impact of the positive factors of the airline cabin crew system on job satisfaction, team capability, and the mental health of cabin crew members. In future studies, we hope that other negative factors, besides the positive factors of the cabin crew team system, will be examined further so that plans for the efficient operation and improvement of the cabin crew system can be formulated through the verification of problems within the domestic airline cabin crew system. Finally, as team systems have generally been introduced in large domestic airlines, the case of foreign airlines that do not operate using the team system should also be reviewed to provide clear guidelines for the eventual introduction and sustainable development of the team system.

## Figures and Tables

**Table 1:** Characteristics of the sample.

Variables	Index	Frequency (*n*)	Percent (%)	Mean (SD)
Gender	Female	234	86.7	
	Male	36	13.3	
Age	20–25	5	1.9	33.44(5.38)
	26–30	92	34.1	
	31–35	84	31.1	
	36–40	64	23.7	
	41–45	18	6.7	
	over 45	7	2.6	
Marital Status	Single	160	59.3	
	Married	110	40.7	
Education	College degree	55	20.4	
	University degree	198	73.3	
	In graduate school	11	4.1	
	Graduate degree	6	2.2	
Working Period	Less than 2 years	7	2.6	8.50(5.23)
	2–5 years	55	20.4	
	5–10 years	97	35.9	
	10–15 years	81	30.0	
	Over 15 years	30	11.1	
Working Grade	Attendant	134	49.6	
	Assistant Purser	91	33.7	
	Purser	38	14.1	
	Senior Purser	5	1.9	
	Chief Purser	2	0.7	
Average Annual Income	Less than KRW 20 million	2	0.7	
	KRW 20–35 million	50	18.5	
	KRW 35–50 million	114	42.2	
	Over KRW 50 million	104	38.5	
Working Position	Manager	13	4.8	
	Assistant Manager	12	4.4	
	Associate	245	90.7	
Total		270	100.0	

**Table 2:** Results of confirmative factor analysis and confidence analysis.

Factor	Question	M(SD)	Loading	Cronbach’s α
Sense of Belonging (4)	1. I feel that I am an important member in our team.	3.60 (0.70)	0.871	0.873
	2. My team’s success is considered my success.	3.48 (0.78)	0.799	
	3. I offer my help when my team members need it.	3.98 (0.68)	0.745	
	4. I am willing to make sacrifices for my team.	3.47 (0.68)	0.774	
Mutual Support (3)	1. My team members encourage each other and work in a cooperative atmosphere.	3.99 (0.65)	0.832	0.865
	2. My team members share necessity data, ideas, and information for work.	4.01 (0.69)	0.831	
	3. My team members are able to monitor the progress of other team members during the flight and help with undesignated duties.	4.07 (0.64)	0.816	
Communication (4)	1. My team members have the tendency to communicate actively with each other.	3.69 (0.64)	0.814	0.872
	2. I prefer to make decisions through discussions with other team members when making uncertain or difficult decisions.	3.89 (0.69)	0.761	
	3. My team members exchange the necessary information for smooth and accurate work.	3.79 (0.66)	0.833	
	4. My team members have the tendency to express their thoughts and opinions freely about work.	3.51 (0.76)	0.776	
Motivation (5)	1. The reason I work hard is to be recognized by my managers and team members.	3.50 (0.82)	0.629	7.95
	2. The reason I work hard is to receive a good evaluation that directly relates to my promotion opportunities (salary increase).	3.76 (0.68)	0.677	
	3. The reason I work hard is to feel a sense of accomplishment for my improved work skills and knowledge about in-flight duties.	3.69 (0.68)	0.595	
	4. The reason I work hard is because this job suits my aptitude and is enjoyable.	3.65 (0.76)	0.590	
	5. The reason I work hard is because I am able to help my team without any inconvenience.	3.90 (0.64)	0.851	
Work Flexibility (5)	1. When working as a team, it is easier to accomplish in-flight duties.	3.88 (0.87)	0.866	0.913
	2. When working as a team, I am able to respond more flexibly to customer requests.	3.76 (0.87)	0.807	
	3. When encountering new tasks or taking on tasks in a difficult situation, the flight team makes it easier to adapt to the tasks.	3.89 (0.80)	0.863	
	4. The flight team enables me to learn or acquire tasks at a faster speed.	3.90 (0.81)	0.839	
	5. As each member’s capabilities and tasks are familiar, it is relatively easy to help them or take their place.	3.92 (0.78)	0.754	
Job Satisfaction (4)	1. I am satisfied with my work in general.	3.36 (0.74)	0.881	0.918
	2. I am satisfied with my relationship with my manager and teammates.	3.31 (0.74)	0.870	
	3. My current salary is at a satisfactory level compared to other companies and those with the same position.	3.20 (0.81)	0.883	
	4. Our company’s promotion opportunities are at a satisfactory level compared to other companies.	2.92 (0.87)	0.815	
Team Potency (4)	1. Overall, our team performs in-flight duties easily and well.	3.77 (0.71)	0.814	0.891
	2. Our team performs teamwork very well.	3.71 (0.79)	0.901	
	3. Our team feels that we can satisfactorily resolve the conflicts and problems that occur during flight operations.	3.65 (0.82)	0.844	
	4. Our team has excellent capabilities and achieves high performance.	3.51 (0.80)	0.721	
Mental Health (5)	1. During team flight briefings, I feel comfortable and can concentrate on my duties easily.	3.69 (0.94)	0.852	0.928
	2. During team flight briefings, I become interested in my duties.	3.43 (0.95)	0.851	
	3. When flying as a team, I feel more comfortable and find it easier to concentrate on my duties.	3.70 (0.91)	0.898	
	4. When flying as a team, I become more interested in my duties.	3.50 (0.90)	0.875	
	5. When flying as a team, I feel more comfortable and pleased during the overseas staying period.	3.57 (0.99)	0.783	

Note: χ^2^ = 806.521(df = 499, *p* < 0.001), CFI = 0.953, TLI = 0.947, RMSEA = 0.048.

**Table 3:** Convergence feasibility validation.

Variations	Composite Reliability (CR)	Average Variance Extracted (AVE)
Sense of Belonging	0.933	0.779
Mutual Support	0.937	0.833
Communication	0.936	0.784
Motivation	0.915	0.686
Work Flexibility	0.941	0.762
Job Satisfaction	0.948	0.821
Team Potency	0.932	0.774
Mental Health	0.937	0.749

**Table 4:** Verification validation.

Variations	1	2	3	4	5	6	7	8
1. Sense of Belonging	(0.779)	0.336	0.266	0.336	0.237	0.262	0.237	0.253
2. Mutual Support	0.580	(0.833)	0.529	0.206	0.384	0.100	0.415	0.326
3. Communication	0.516	0.727	(0.784)	0.178	0.309	0.156	0.454	0.386
4. Motivation	0.580	0.454	0.422	(0.686)	0.241	0.138	0.108	0.204
5. Work Flexibility	0.487	0.620	0.556	0.491	(0.762)	0.148	0.460	0.659
6. Job Satisfaction	0.512	0.317	0.395	0.371	0.385	(0.821)	0.217	0.198
7. Team Potency	0.487	0.644	0.674	0.328	0.678	0.466	(0.774)	0.543
8. Mental Health	0.503	0.571	0.621	0.452	0.812	0.445	0.737	(0.749)

Note: diagonal: (AVE), diagonal down: correlation coefficient, diagonal up: square of correlation coefficient (determination coefficient).

**Table 5:** Verification validation.

Variations	Range	Average	Standard Deviation	Skewness	Kurtosis
Sense of Belonging	1–5	3.63	0.61	−0.16	0.03
Mutual Support	1–5	4.02	0.58	−0.47	0.63
Communication	1–5	3.72	0.59	−0.05	0.04
Motivation	1–5	3.70	0.53	−0.06	0.08
Work Flexibility	1–5	3.87	0.71	−0.77	1.37
Job Satisfaction	1–5	3.20	0.71	0.35	−0.01
Team Potency	1–5	3.66	0.68	−0.42	1.13
Mental Health	1–5	3.58	0.83	−0.40	0.14

**Table 6:** Fit of structural equation model.

χ^2^	df	*p*	CFI	TLI	RMSEA
820.832	500	<0.001	0.951	0.945	0.049
